# Tribo-Mechanical Properties of HVOF Deposited Fe_3_Al Coatings Reinforced with TiB_2_ Particles for Wear-Resistant Applications

**DOI:** 10.3390/ma9020117

**Published:** 2016-02-19

**Authors:** Mahdi Amiriyan, Carl Blais, Sylvio Savoie, Robert Schulz, Mario Gariépy, Houshang Alamdari

**Affiliations:** 1 Département de génie des mines, de la métallurgie et des matériaux, Université Laval, Québec, QC G1V 0A6, Canada; Mahdi.amiriyan.1@ulaval.ca (M.A.); carl.blais@gmn.ulaval.ca (C.B.); 2 Aluminium Research Centre-REGAL, Québec, QC G1V 0A6, Canada; 3 Hydro-Quebec Research Institute, Varennes, QC J3X 1S1, Canada; savoie.sylvio@ireq.ca (S.S.); schulz.robert@ireq.ca (R.S.); 4 Weir American Hydro, Weir Canada Inc., LaSalle, QC H8N 1V1, Canada; mario.gariepy@weirgroup.com

**Keywords:** iron aluminide, titanium diboride, high-velocity oxy-fuel, HVOF, mechanical properties, dry sliding wear

## Abstract

This study reveals the effect of TiB_2_ particles on the mechanical and tribological properties of Fe_3_Al-TiB_2_ composite coatings against an alumina counterpart. The feedstock was produced by milling Fe_3_Al and TiB_2_ powders in a high energy ball mill. The high-velocity oxy-fuel (HVOF) technique was used to deposit the feedstock powder on a steel substrate. The effect of TiB_2_ addition on mechanical properties and dry sliding wear rates of the coatings at sliding speeds ranging from 0.04 to 0.8 m·s^−1^ and loads of 3, 5 and 7 N was studied. Coatings made from unreinforced Fe_3_Al exhibited a relatively high wear rate. The Vickers hardness, elastic modulus and wear resistance of the coatings increased with increasing TiB_2_ content in the Fe_3_Al matrix. The wear mechanisms strongly depended on the sliding speed and the presence of TiB_2_ particles but were less dependent on the applied load.

This study reveals the effect of TiB_2_ particles on the mechanical and tribological properties of Fe_3_Al-TiB_2_ composite coatings against an alumina counterpart. The feedstock was produced by milling Fe_3_Al and TiB_2_ powders in a high energy ball mill. The high-velocity oxy-fuel (HVOF) technique was used to deposit the feedstock powder on a steel substrate. The effect of TiB_2_ addition on mechanical properties and dry sliding wear rates of the coatings at sliding speeds ranging from 0.04 to 0.8 m·s^−1^ and loads of 3, 5 and 7 N was studied. Coatings made from unreinforced Fe_3_Al exhibited a relatively high wear rate. The Vickers hardness, elastic modulus and wear resistance of the coatings increased with increasing TiB_2_ content in the Fe_3_Al matrix. The wear mechanisms strongly depended on the sliding speed and the presence of TiB_2_ particles but were less dependent on the applied load.

This study reveals the effect of TiB_2_ particles on the mechanical and tribological properties of Fe_3_Al-TiB_2_ composite coatings against an alumina counterpart. The feedstock was produced by milling Fe_3_Al and TiB_2_ powders in a high energy ball mill. The high-velocity oxy-fuel (HVOF) technique was used to deposit the feedstock powder on a steel substrate. The effect of TiB_2_ addition on mechanical properties and dry sliding wear rates of the coatings at sliding speeds ranging from 0.04 to 0.8 m·s^−1^ and loads of 3, 5 and 7 N was studied. Coatings made from unreinforced Fe_3_Al exhibited a relatively high wear rate. The Vickers hardness, elastic modulus and wear resistance of the coatings increased with increasing TiB_2_ content in the Fe_3_Al matrix. The wear mechanisms strongly depended on the sliding speed and the presence of TiB_2_ particles but were less dependent on the applied load.

This study reveals the effect of TiB_2_ particles on the mechanical and tribological properties of Fe_3_Al-TiB_2_ composite coatings against an alumina counterpart. The feedstock was produced by milling Fe_3_Al and TiB_2_ powders in a high energy ball mill. The high-velocity oxy-fuel (HVOF) technique was used to deposit the feedstock powder on a steel substrate. The effect of TiB_2_ addition on mechanical properties and dry sliding wear rates of the coatings at sliding speeds ranging from 0.04 to 0.8 m·s^−1^ and loads of 3, 5 and 7 N was studied. Coatings made from unreinforced Fe_3_Al exhibited a relatively high wear rate. The Vickers hardness, elastic modulus and wear resistance of the coatings increased with increasing TiB_2_ content in the Fe_3_Al matrix. The wear mechanisms strongly depended on the sliding speed and the presence of TiB_2_ particles but were less dependent on the applied load.

This study reveals the effect of TiB_2_ particles on the mechanical and tribological properties of Fe_3_Al-TiB_2_ composite coatings against an alumina counterpart. The feedstock was produced by milling Fe_3_Al and TiB_2_ powders in a high energy ball mill. The high-velocity oxy-fuel (HVOF) technique was used to deposit the feedstock powder on a steel substrate. The effect of TiB_2_ addition on mechanical properties and dry sliding wear rates of the coatings at sliding speeds ranging from 0.04 to 0.8 m·s^−1^ and loads of 3, 5 and 7 N was studied. Coatings made from unreinforced Fe_3_Al exhibited a relatively high wear rate. The Vickers hardness, elastic modulus and wear resistance of the coatings increased with increasing TiB_2_ content in the Fe_3_Al matrix. The wear mechanisms strongly depended on the sliding speed and the presence of TiB_2_ particles but were less dependent on the applied load.

This study reveals the effect of TiB_2_ particles on the mechanical and tribological properties of Fe_3_Al-TiB_2_ composite coatings against an alumina counterpart. The feedstock was produced by milling Fe_3_Al and TiB_2_ powders in a high energy ball mill. The high-velocity oxy-fuel (HVOF) technique was used to deposit the feedstock powder on a steel substrate. The effect of TiB_2_ addition on mechanical properties and dry sliding wear rates of the coatings at sliding speeds ranging from 0.04 to 0.8 m·s^−1^ and loads of 3, 5 and 7 N was studied. Coatings made from unreinforced Fe_3_Al exhibited a relatively high wear rate. The Vickers hardness, elastic modulus and wear resistance of the coatings increased with increasing TiB_2_ content in the Fe_3_Al matrix. The wear mechanisms strongly depended on the sliding speed and the presence of TiB_2_ particles but were less dependent on the applied load.

## 1. Introduction

During the last few decades, Fe_3_Al and FeAl intermetallics have been of significant interest due to their properties such as relatively low density, relatively high melting point and remarkable resistance to corrosion under sulfidizing and oxidizing environments. Iron aluminides have also been reported to show excellent wetting to carbide and boride phases, making them suitable binders for TiC or TiB_2_-based cermets. However, limited room temperature ductility (less than 5%) and poor wear resistance have been the principal obstacles to the acceptance of iron aluminides in many applications. It seems that the low wear resistance of iron aluminides has a correlation with their hardness and Young’s modulus. Microstructures containing second-phase hard particles can exhibit higher dry sliding wear resistance compared to single-phase materials [1,2]. Thus, in the case of an iron aluminide/ceramic composite, combined properties of the matrix and ceramic component may result in hardness increase and, consequently, higher wear resistance.

Recently, there has been considerable interest in the development of iron aluminide coatings reinforced with suitable type and volume fraction of ceramic particles. These composite coatings are designed to protect the substrate from aggressive environments and increase the life span of the underlying material. Some studies have also shown that coating structural materials is an effective method to overcome difficulties in fabrication and shaping of iron aluminide/ceramic composites [1,3]. The high-velocity oxy-fuel (HVOF) spraying technique is one of the potential approaches for producing these coatings with thicknesses of a few hundred microns [4]. The HVOF has widely been used in many industries as well as research works due to its cost effectiveness and high flexibility. Often, the HVOF is used to deposit coatings to protect a substrate against wear, oxidation and corrosion. Higher deposition velocity and lower process temperature have been reported to result in limited decomposition of ceramic particles during the HVOF deposition [5]. In addition, coatings with low porosity, high hardness, high yield stress [6], improved adhesion to substrate [7] and compressive residual stresses [4] are the main advantages of the HVOF over other thermal spray techniques. The HVOF yields a smoother as-sprayed surface because of higher impact energies, resulting in production of higher wear-resistant coatings [8]. 

Although there have been some indications in the literature on the efficiency of hard ceramic particles on enhancing the mechanical and tribological properties of iron aluminide matrix, there is a limited number of studies that aim to elucidate the role of the ceramic in theses coatings. Chen *et al.* [9] noted that FeAl intermetallics, having ceramic reinforcements, possessed excellent sliding wear resistance. Zhang *et al.* [10] found similar results on the effect of TiC content on dry sliding wear behavior of the Fe_3_Al-TiC composites. More recently, Amiriyan *et al.* [11] studied the *in-situ* formation of TiC nano-particles in iron aluminide matrix and the effect of this ceramic on hardness and sliding wear behavior of the coatings. In the current paper, the effect of different quantities of pre-formed TiB_2_ (30 and 50 vol.%) in Fe_3_Al matrix on microhardness, elastic modulus and tribological properties of coatings deposited using the HVOF technique is investigated.

## 2. Materials and Methods

The starting Fe_3_Al powder with 2 at.% Cr (added to improve mechanical properties) was purchased from Ametek Ltd., Eighty Four, PA, USA. Highly pure titanium diboride powder was added as reinforcing secondary phase at two different volume fractions, namely 30% and 50%. The TiB_2_ powder was milled with the iron aluminide powder in a high energy ball mill (Zoz GmbH, Wenden, Germany) using steel balls and crucible as the milling media. To prevent oxidation, the milling process was carried out under an argon gas flow. The powders were milled for 3 h and then discharged and sieved through 270 and 625 mesh (53 and 20 μm, respectively) sieves in order to obtain a ready-to-deposit Fe_3_Al-TiB_2_ composite powder.

Thermal spraying was carried out using a TAFA HVOF system from Praxair (Concord, NH, USA). The spraying parameters, detailed in Table 1, were selected based on a previous investigation on the influence of various parameters on deposition efficiencies. Mild steel plates (grade AISI 1020 with dimensions of 190 mm × 120 mm × 5 mm) were used as substrate material. The substrates were sandblasted on one side and washed with acetone and ethanol, respectively, and then fixed on a stationary support, perpendicular to the gun. The HVOF gun was moved in *x*–*y* direction in order to cover the entire surface of the substrate.

Friction and dry sliding wear tests were carried out according to the ASTM G99-05 pin-on-disk standard method. The surface of the coated samples (initially having a roughness of *R*_a_ ≈ 10–20 μm) was ground using abrasive silicon carbide (SiC) papers of 180, 240, 320 and 600 grades successively, followed by polishing with 6-μm and 1-μm diamond paste and, finally, a 0.5-μm alumina solution in order to obtain a surface finish of *R*_a_ ≤ 1 μm.

Prior to the sliding tests, the polished samples were cleaned in an ultrasonic cleaner with acetone and ethanol, respectively. Sliding wear tests were performed in a UMT tester (Bruker Corporation, San Jose, CA, USA) under a controlled environment at ambient conditions of temperature (~25 °C) and humidity (~30%). The tests were conducted under an applied load ranging from 3 to 7 N and at a sliding speed ranging from 0.04 to 0.8 m·s^−1^ over a sliding distance of 1000 m. After each test, the depth and the width of each wear scar were measured at 4 different equidistant points using a Dektak-150 surface profilometer (Bruker Corporation, San Jose, CA, USA). The average of these values was considered as the wear track cross section area. The wear volume of each specimen was calculated by multiplying the average wear area to the circumference and normalized against load and distance, based on Equation (1).

(1) *K* = *V*/*w*·*s*

where *V* (mm^3^) is the worn volume, *w* (N) is the normal load, and *s* (m) is the total sliding distance. For each experiment, a new 6.33-mm diameter alumina ball with a Vickers hardness of 1600–1700 H_v_ was used as the counterpart. The tests for each composition were conducted on three different samples under the same conditions to ensure repeatability of the results. However, all coatings were produced using the same feedstock powder for each composition.

The phase content of the coatings was investigated using an X-ray diffractometer (SIEMENS, D5000, Karlsruhe, Germany), equipped with CuK_α_ radiation. A scanning electron microscope (JEOL, 840, Tokyo, Japan) was used to observe the coating cross sections and morphology of the wear tracks, wear debris, and alumina counterpart. Microhardness measurements were performed on the polished cross section of each coating by Vickers indentation (LECO, M-400FT, St. Joseph, MI, USA) at a load of 200 gf and a dwell time of 15 s. For each sample, the average of twelve indentations at different points was calculated and reported as the hardness value.

The microindentation tests were carried out on cross section of the coatings using a microindentation apparatus (CSM, Peseux, Switzerland), equipped with a Vickers tip and software to analyze the hardness and elastic modulus. The coatings were tested to a maximum load of 500 mN with a loading time of 15 s. The entire loading/unloading process was recorded, resulting in a load *versus* displacement curve. All data were analyzed according to the standard Oliver-Pharr procedure [12]. The reported value is the average of 50 indentations for each specimen. A large distance of 200–250 μm between the indentations was chosen in order to avoid interaction between the strain hardening regions or any possible microcrack caused by the indentation.

## 3. Results and Discussion

**Figure 1:**
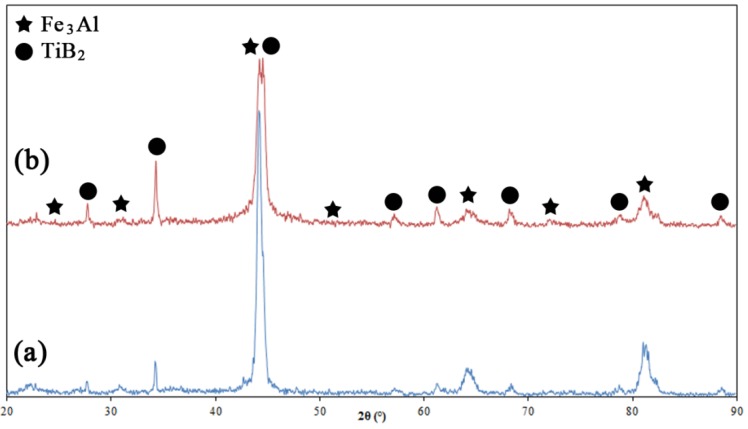
XRD patterns of the (**a**) Fe_3_Al-30 vol.% TiB_2_ and (**b**) Fe_3_Al-50 vol.% TiB_2_ HVOF composite coatings.

**Figure 2:**
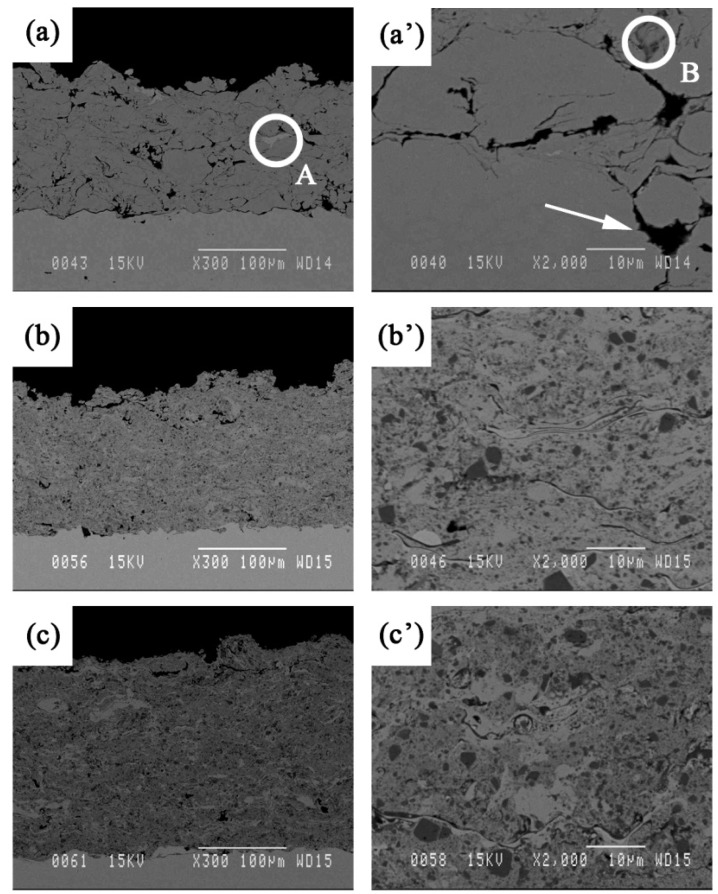
Cross-sectional backscattered SEM images of the unreinforced (0 vol.% TiB_2_) Fe_3_Al (**a** and **a’**), 30 (**b** and **b’**) and 50 (**c** and **c’**) vol.% TiB_2_ coatings. The arrow on a’ indicates a pore.

The phase composition of the as-sprayed coatings did not differ significantly from that of the starting powders. Figure 1 shows the XRD patterns of the HVOF deposited composite coatings. The samples mostly consist of a mixture of Fe_3_Al and TiB_2_ phases with no noticeable presence of other phases. Such a result was expected due to the fact that high particle velocity and lower flame temperature in the HVOF would prevent the decomposition of the feedstock powder during the process. In a recent paper, Chen *et al.* [13] reported that a trace of titanium oxide (as a result of TiB_2_ oxidation) was found in TiB_2_ reinforced HVOF composite coatings. However, these researchers claimed that such decomposition is small, since TiB_2_ particles are quite stable and do not melt during the HVOF process. Figure 2 shows the cross-sectional backscattered SEM images of the coatings. The images reveal highly compacted and uniform lamellar microstructures with an average thickness of about 150 to 200 μm and a porosity around 2%–4% (measured by image analysis). The images also reveal the presence of defects such as inter-splat micro-pores (see Figure 2a’).

**Figure 3:**
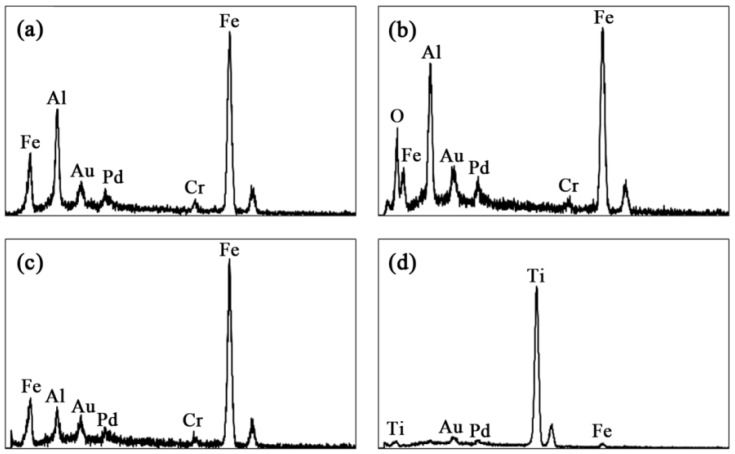
EDS spectra of the (**a**) main grey phase in Fe_3_Al coating, (**b**) dark grey oxide inclusions, (**c**) light grey areas and (**d**) TiB_2_ particles.

Figure 2a’–c’ show cross-sectional morphologies of the coatings at higher magnifications. Different contrasts are observed in these images alongside with porosities. The EDS (Energy-Dispersive Spectroscopy) patterns of each region are shown in Figure 3. According to these EDS patterns, the dark grey regions (a sample of which is indicated by B in Figure 2a’), encountered at the splat boundaries, are rich in oxygen and therefore correspond most likely to oxide inclusions enriched in Al compared to the iron aluminide matrix (Figure 3b). The light grey regions (indicated by A in Figure 2a) are rich in Fe (Figure 3c). These regions are most likely due to Fe contamination of the powder by the high energy ball milling process or may come from Al-depleted iron aluminide particles due to Al oxidation during the spray process. Figure 3d shows that the TiB_2_ particles (dark grey regions as well) are well distinguishable and are thoroughly dispersed into the iron aluminide matrix (round particles in Figure 2b’,c’).

**Figure 4:**
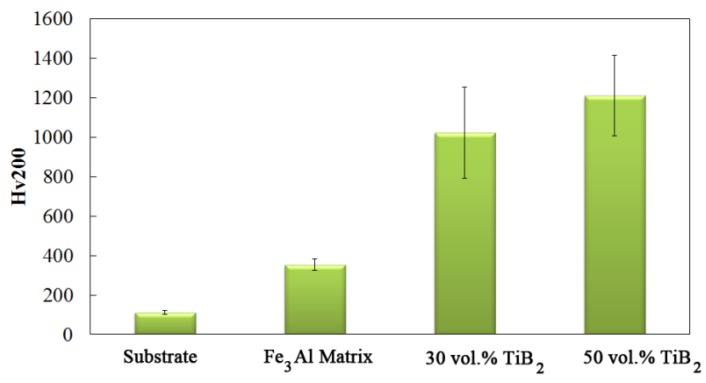
Hardness of the substrate and coatings as a function of different TiB_2_ content.

Figure 4 shows the Vickers hardness (H_v200_) of the substrate and the coatings with different TiB_2_ contents. Very low hardness value was observed for the Fe_3_Al coating. As expected, the hardness is strongly affected by the TiB_2_ content in the matrix. It is clear that the addition of titanium diboride significantly increases the Vickers hardness. A very high hardness value of about 1200 was observed for the 50 vol.% TiB_2_ sample. This value is three times greater than that of the Fe_3_Al coating. One can observe that the error bar in the Vickers hardness is much larger for the composite samples. This might be as a result of microstructural inhomogeneity at small length scale, since the indentation section is too small, being in the range of dispersion inhomogeneity. According to Melendez and McDonald [14], it is common for such coatings to exhibit high standard deviations due to non-homogeneous distribution of reinforcing particles. The Vickers hardness measurement depends on whether the indenter is located on areas with a high concentration of TiB_2_ reinforcement or on areas with a high concentration of iron aluminide matrix.

The wear behavior of materials has long been related to their hardness. Elastic modulus, however, has been reported to have a significant influence on tribological behavior. Leyland & Matthews [15] clarified the merit of *H*/*E* ratio (hardness/elastic modulus) on controlling wear properties of coatings. Hard materials generally exhibit a high elastic modulus, which may have undesirable effects on wear behavior. Therefore, consideration of coating hardness and elastic modulus is important in order to understand the mechanical and tribological behaviors of coatings.

**Figure 5:**
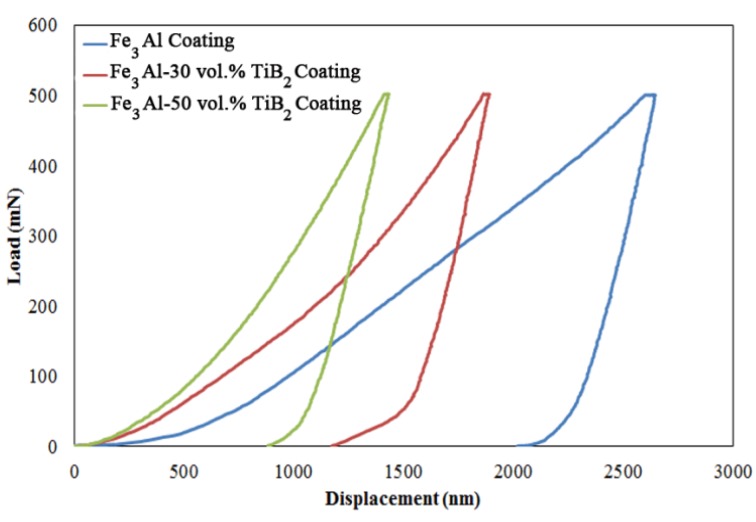
Typical load-displacement curves of the coatings.

Typical load-displacement curves for Fe_3_Al and Fe_3_Al-TiB_2_ composite coatings are presented in Figure 5. The average results from all microindentation tests are indicated in Table 2. In Figure 5, it can be seen that as the TiB_2_ fraction increases, the displacement and indentation depth decrease. In addition, the hardness and the elastic modulus of the coatings increase as well as the *H*/*E* ratios with the addition of ceramic particles. The hardness and elastic modulus of the Fe_3_Al-based coatings increase by more than 300% and 200% respectively due to the addition of TiB_2_ particles. This enhancement can be attributed to the strengthening effects of well-dispersed hard TiB_2_ particles with strong interfacial bonds in the iron aluminide matrix. Xu and Liu [2] have investigated the effect of *in-situ* synthesized TiB_2_ particulates on the wear characteristic of the Fe-Al-Ti-B system, and similar increases of hardness and elastic modulus have been reported.

**Figure 6:**
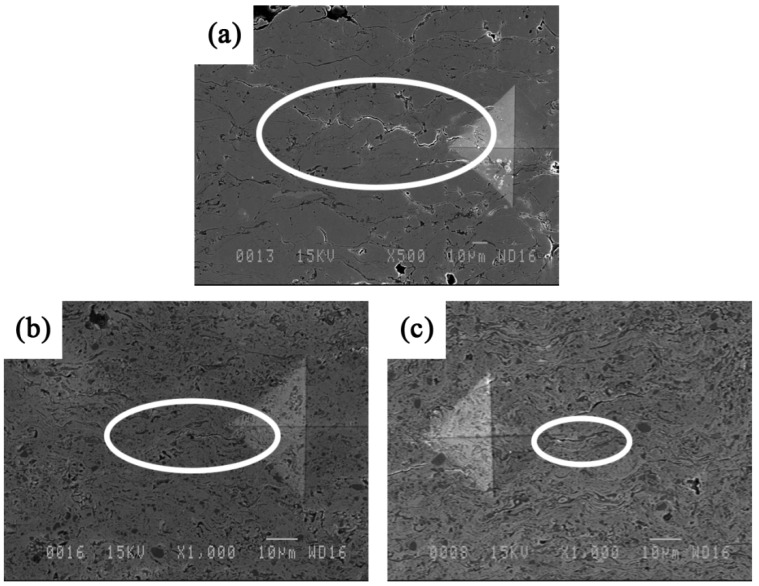
SEM images of Vickers indentation cracks (induced at high load of 1000 gf) on (**a**) Fe_3_Al, (**b**) Fe_3_Al-30 vol.% TiB_2_ and (**c**) Fe_3_Al-50 vol.% TiB_2_ coating cross sections. Crack propagation paths have been indicated by white ovals.

In thermal sprayed coatings, the preferential propagation path of cracks is mostly parallel to the substrate [16,17]. SEM images in Figure 6 show the crack propagation in a cross section of coatings due to Vickers indentations. In Fe_3_Al, cracks propagate along the weak inter-splat interfaces. The crack in Figure 6a for the Fe_3_Al appears to be quite long (>100 µm) compared to those observed in the composite specimens (Figure 6b,c). Crack deflections as a result of the presence of TiB_2_ particles may be a reason for the enhancement of the mechanical properties of such composite materials.

**Figure 7:**
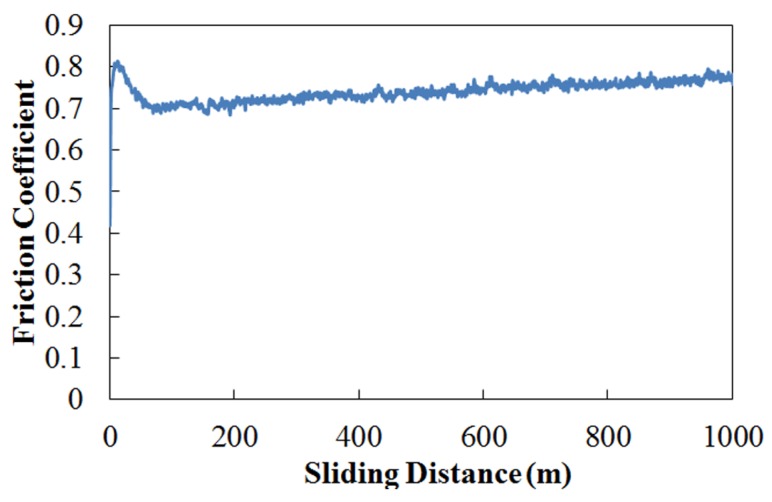
Typical friction coefficient curve for a thermally sprayed Fe_3_Al coating.

**Figure 8:**
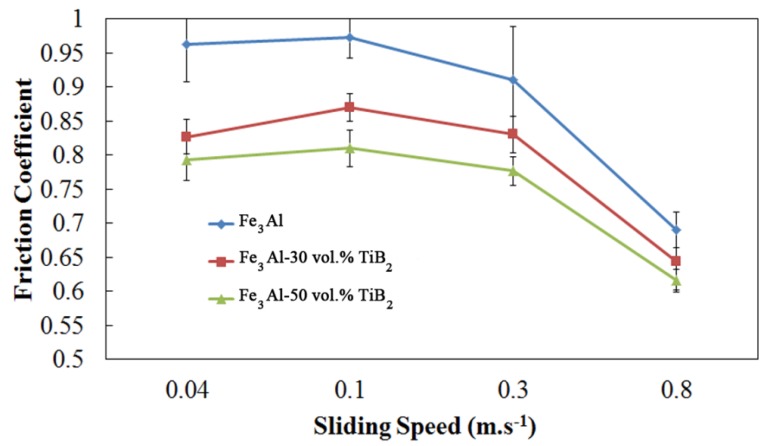
Friction coefficient *versus* sliding speed at the applied load of 5 N.

Figure 7 is a typical curve of the friction coefficient *versus* sliding distance for Fe_3_Al coatings against an alumina counterpart under dry sliding condition. Alumina has been chosen as counter material in this study to compare results with other materials under investigation at Hydro-Quebec Research Institute. An initial rise is observed, followed by a fall and a steady state after a certain sliding distance. The initial rise in the friction coefficient (static friction coefficient) has been explained to be either the result of the high adhesive contact between the counterpart and the coating surface [1,18,19] or from the surface roughness and early anchoring between the asperities of the counterpart and the coating [20]. In the present study, adhesion is most likely the dominant effect since the samples have been polished to mirror finish prior to conduct the experiments. The variation of the friction coefficient with TiB_2_ content and sliding speed is shown in Figure 8. The friction coefficient decreases by increasing the TiB_2_ volume fraction in the coating. As the fraction of TiB_2_ increases, the number of interfaces between Fe_3_Al and alumina decreases, and the number of interfaces between TiB_2_ and the counter material increases. This leads to a decrease in the friction coefficient probably because of a reduction in the adhesion between materials.

The friction coefficients of Fe_3_Al and composite specimens show a relatively stable trend with increasing sliding speed up to 0.3 m·s^−1^ and then decrease at higher speed. High sliding speed often leads to local heating and surface oxidation [21]. The changes in the nature and morphology of the surface at high speed, which may lead to a reduced contact area, decrease the friction coefficient and the wear of the components, as will be discussed in the next section.

**Figure 9:**
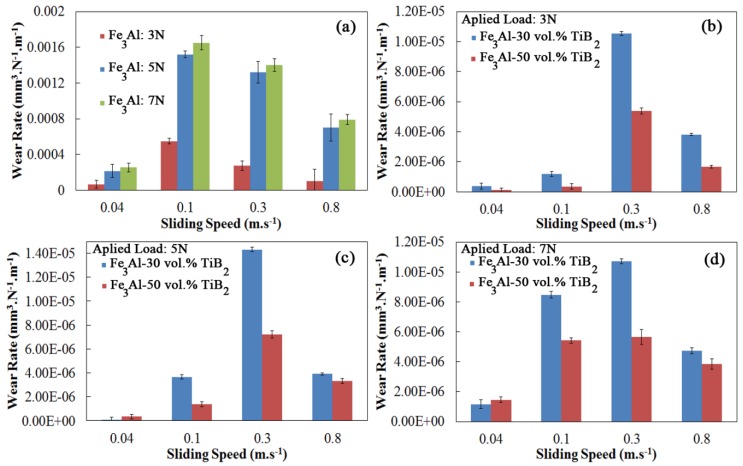
Wear rates as a function of sliding speed and applied load. (**a**) Fe_3_Al coatings and (**b**–**d**) composite coatings.

The effect of titanium diboride reinforcing particles on dry sliding wear rates is shown in Figure 9. As expected, the wear rates of the composite coatings are considerably lower than that of Fe_3_Al coating under all sliding conditions.

The wear rate of the Fe_3_Al coatings is on the order of 10^−3^ to 10^−4^ mm^3^·N^−1^·m^−1^. On the other hand, the wear rates of the 50 vol.% TiB_2_ coating is 1 to 3 orders of magnitude lower. This improvement is attributed to the increase in Vickers hardness and *H*/*E* values, caused by the presence of TiB_2_ particles, and is in agreement with Archard’s law, which relates wear of materials to hardness [22]. A similar improvement in properties has been previously reported in other systems [9,23,24,25]. Furthermore, surface contact between sample and counterpart is controlled by the amount of reinforcing particles in the composite as mentioned before. In the case of composite coatings, the fraction of soft matrix in direct contact with the hard counterpart (alumina ball in this case) is less and this contributes to the reduction in friction coefficients and wear rates.

In addition to the volume fraction of TiB_2_ particles, the wear also depends on sliding speed. Figure 9 shows the effect of the sliding speed on wear rates of coatings. In the case of the unreinforced Fe_3_Al coatings, increasing the sliding speed from 0.04 to 0.1 m·s^−1^ resulted in a large increase in wear rates. With a further increase in sliding speed, the wear rate decreases, thus producing a maximum at the speed of 0.1 m·s^−1^. By incorporation of the TiB_2_ particles within the matrix, the critical sliding speed, corresponding to the maximum wear rate, is shifted to higher speeds. The occurrence of such a maximum in wear rates suggests that there is a change in wear mechanisms with sliding speeds. A similar rise in wear rates at low sliding speeds has been previously reported in such materials during tribological tests. Guan *et al.* [26] suggested that the increase in wear rates with sliding speed is due to the delamination of surface layers.

One observes in Figure 9 that the minimum values of wear rate occur at low sliding speeds and low applied loads. The rates rise at higher loads, and intermediate velocities, by further increasing the speed, therefore decrease, as in the case of the friction coefficient. The wear rate presents a higher dependence on the sliding speed than on the applied load. In fact, the wear rate seems to be nearly independent of the load at very fast sliding speeds (*i.e.*, 0.8 m·s^−1^).

**Figure 10:**
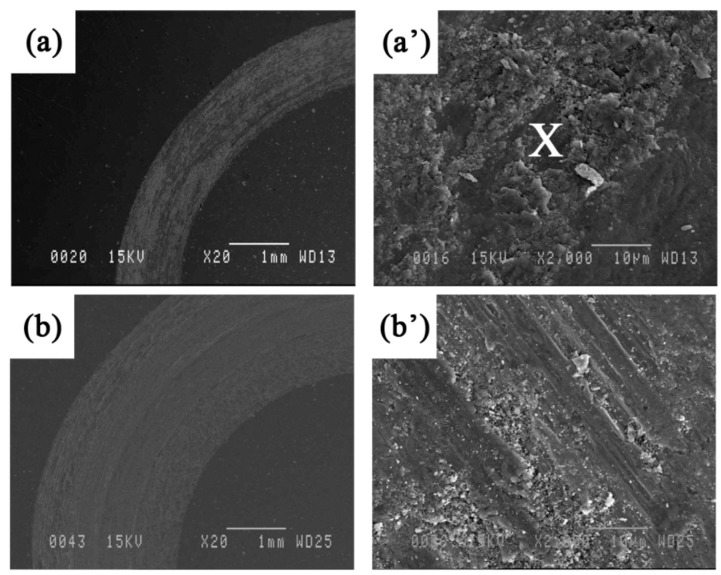

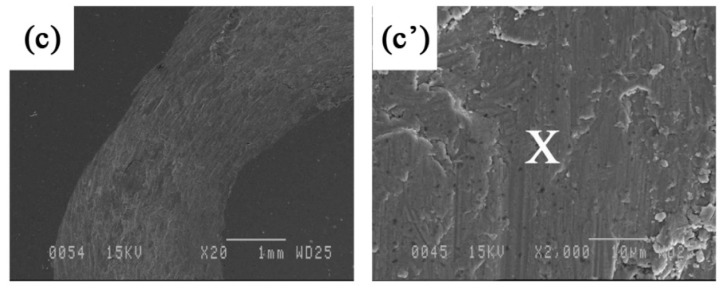
SEM images at different magnifications of the worn surfaces for Fe_3_Al at a sliding speed of (**a** and **a’**) 0.04, (**b** and **b’**) 0.1 and (**c** and **c’**) 0.8 m·s^−1^ under a load of 5 N. EDS was taken from the points marked by X.

To identify predominant wear mechanisms in each operating regime, the surface of wear tracks, wear debris, and the surface of the alumina counterpart have been examined by SEM and analyzed by the EDS technique. Selected SEM images of the wear tracks (at the end of the sliding process, 1000 m length) for Fe_3_Al coatings at different sliding speeds under a load of 5 N are shown in Figure 10. The worn surface consists of wear debris and plowing marks. These worn track features are observed at any load, and the higher the load, the more scratches, and the deeper and wider the wear tracks. These defects have been created by the plowing action of the alumina counterpart while sliding. One observes that, at high speed, the edges of the wear track are not well defined (Figure 10c), suggesting reduced contact areas at some points along the wear track. Small cavities are also observed on the surface at the highest speed. These cavities may come from revealed internal porosities resulting from surfaces in contact moving at high speed.

**Figure 11:**
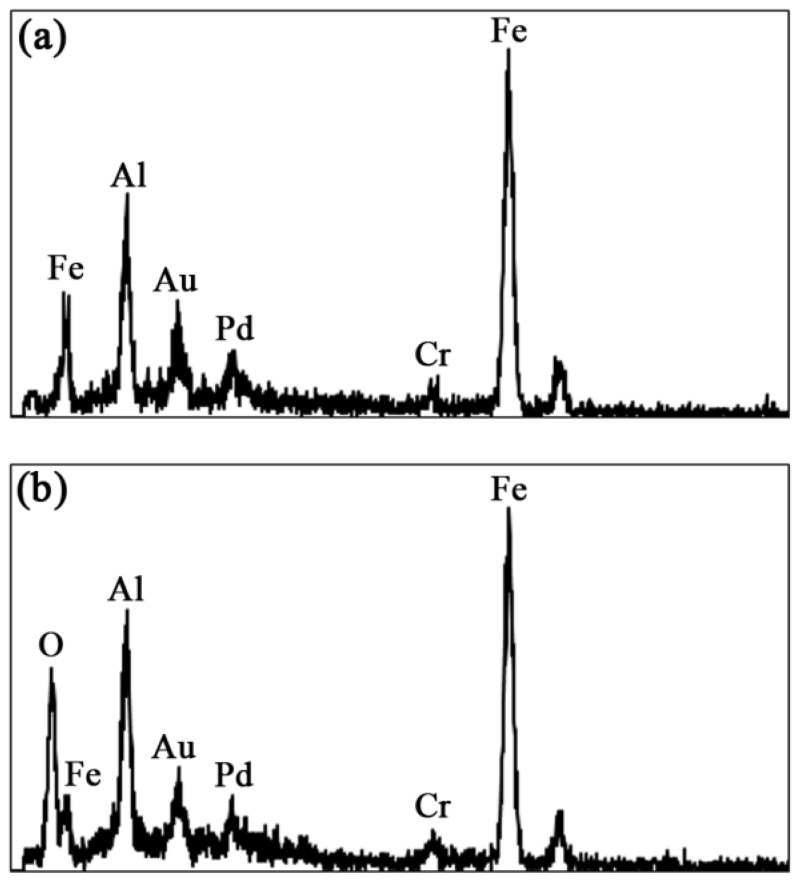
EDS spectra of the worn surface of Fe_3_Al coating at (**a**) 0.04 and (**b**) 0.8 m·s^−1^.

Figure 11 shows the EDS spectra taken inside the wear track at the lowest and highest speeds, respectively. At the lowest speed, only the Fe, Al and Cr peaks from the metal matrix are observed as expected. The Au and Pd peaks come from sample preparation. However, at the highest speed, a strong oxygen peak is seen confirming the oxidation process discussed previously.

**Figure 12:**
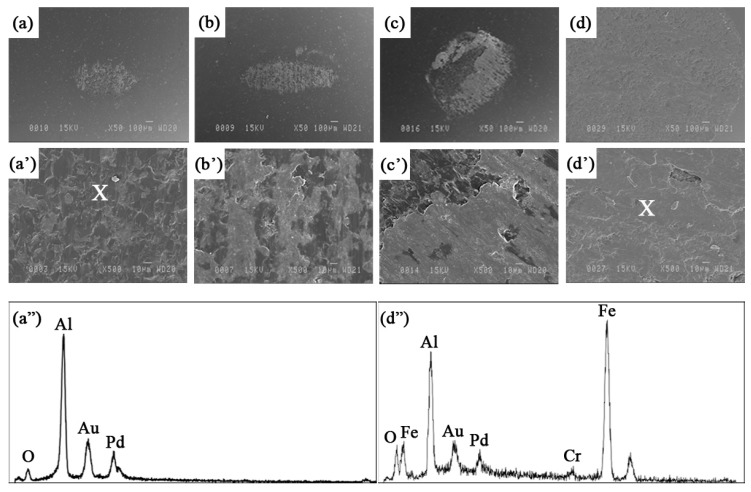
SEM images at different magnifications of the alumina counterpart used against Fe_3_Al at a sliding speed of (**a** and **a’**) 0.04, (**b** and **b’**) 0.1, (**c** and **c’**) 0.3 and (**d** and **d’**) 0.8 m·s^−1^; (**a”**) EDS spectrum of the alumina ball and (**d”**) EDS spectrum of the transferred layer obtained at the highest speed under a load of 5 N. EDS was taken from the points marked by X.

Figure 12 shows images of the alumina counterpart after wear tests at different sliding speeds. The pictures of the transferred layers show that the adhesion of the iron aluminide to the counterpart is significant, indicating that adhesion plays a role in the wear mechanisms of the unreinforced coating. The oxygen peak observed in the EDS spectrum (taken from the point marked by X) of the transferred layer obtained at high speed is consistent with the previous discussion.

Wang *et al.* [21] have calculated an increase of 200 °C in the interfacial temperature when the sliding speed reaches 0.39 m·s^−1^. Yang *et al.* [27] reported that the wear resistance is improved in some systems at high sliding speed because of the formation of an oxide layer. The partially oxidized transferred layer shown in Figure 12d’,d” may act as a lubricant and lead to a reduction of the friction coefficient and wear rate at high speed.

As shown in Figure 4 and Table 2, the microhardness and *H*/*E* ratio of Fe_3_Al are relatively low. Therefore, during the sliding wear of Fe_3_Al, soft material is easily removed because of adhesion and the plowing action of the hard counterpart. As the speed increases, the loading cycle increases and fatigue wear becomes the dominant wear mechanism [27]. Cracks are generated and propagate along inter-splat boundaries, as in the case shown in Figure 6a. This leads to delamination of the coating.

**Figure 13:**
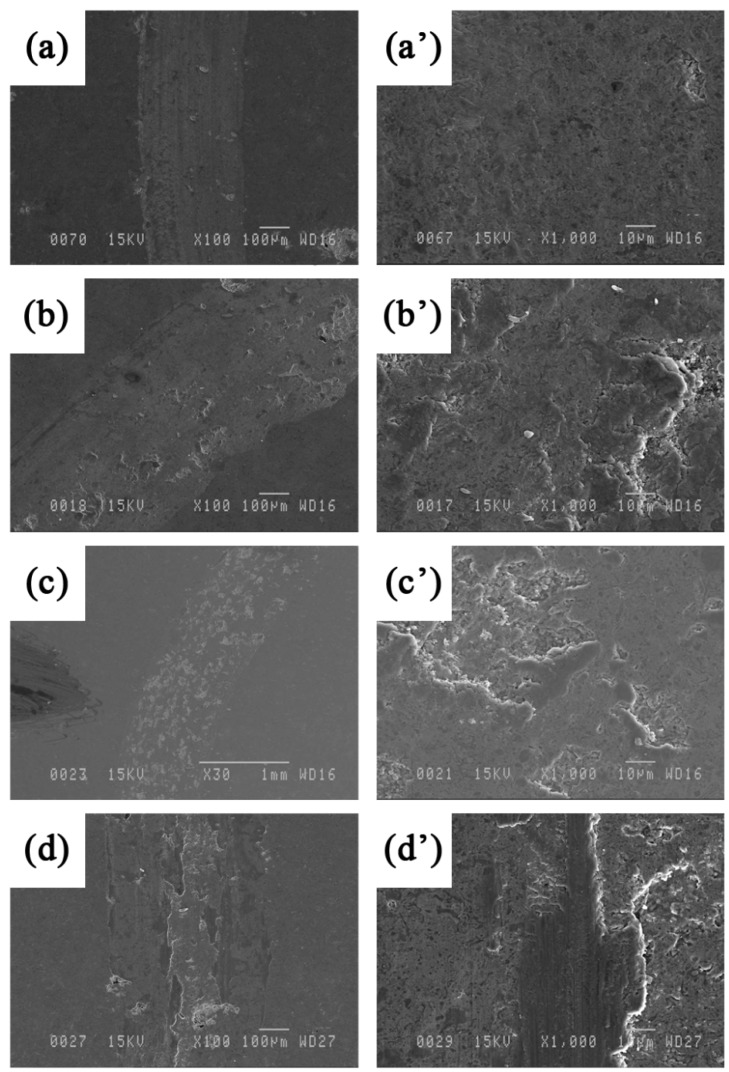
SEM images at different magnifications of the worn surfaces for Fe_3_Al-50 vol.% TiB_2_ at a sliding speed of (**a** and **a’**) 0.04, (**b** and **b’**) 0.1, (**c** and **c’**) 0.3 and (**d** and **d’**) 0.8 m·s^−1^ under a load of 5 N.

**Figure 14:**
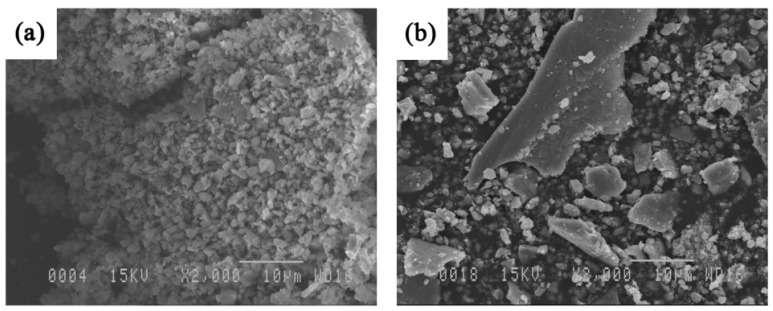
Microscopic morphologies of the wear debris of the Fe_3_Al-TiB_2_ composite coatings worn at sliding speed of (**a**) 0.04 and (**b**) 0.3 m·s^−1^.

Pictures of the wear tracks of the composite coatings are shown in Figure 13 at speeds of 0.04–0.8 m·s^−1^ under the same load of 5 N. No significant wear was found on the surface of the coating when sliding at very low speed (0.04 m·s^−1^). Figure 13b,c show the worn surface at intermediate speeds. The presence of delamination is observed especially in Figure 13c’. SEM analyses of the wear debris (Figure 14) show that the debris at low sliding speed (0.04 m·s^−1^) look like fine particles, while flakes are also observed at higher speed (0.3 m·s^−1^) when maximum wear takes place. It is likely that these flakes peel off from the surface following fatigue cycles. The particles and flake-like debris are similar to those observed by Xu *et al.* [1] in the Fe-Al and Fe-Al/WC thermally sprayed coating. Very small cavities are also observed at high magnification in Figure 13. In this case, in addition to internal porosities, the cavities probably come from hard particle removal from the soft iron aluminide intermetallic matrix surface that binds the reinforcing agent. This leads to the formation of debris shown in Figure 14a. These particles certainly act as a third body during wear; therefore, abrasive wear also plays an important role in the case of the composite coatings. Similar findings were observed in the case of the Fe_3_Al-TiC system studied by the authors recently [11]. As before, increasing the sliding speed promotes the increase of temperature at the interface, which leads to a decrease in the friction coefficient (Figure 8) and wear rates (Figure 9c). Oxidation wear becomes an important wear mechanism at the highest speeds.

**Figure 15:**
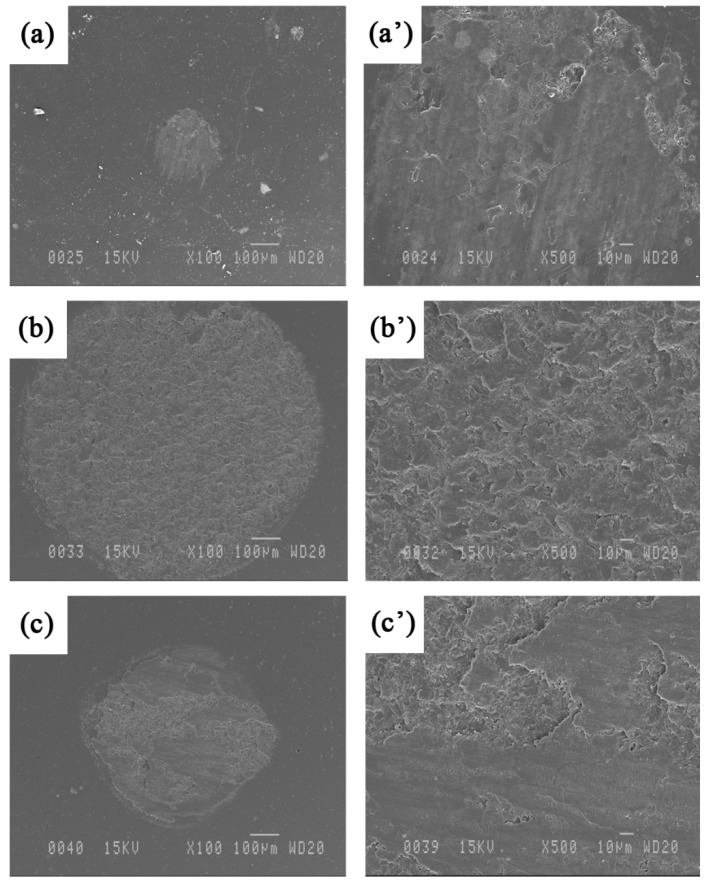
SEM images at different magnifications of the alumina counterpart used against Fe_3_Al-50 vol.% TiB_2_ at a sliding speed of (**a** and **a’**) 0.04, (**b** and **b’**) 0.3 and (**c** and **c’**) 0.8 m·s^−1^ under 5 N load.

SEM images of the alumina counterpart used against composite coatings are shown in Figure 15. Worn regions can be observed on the surface of the alumina ball because of the relatively high hardness of the reinforced coatings. The wear of the alumina ball is significantly larger in this case compared to unreinforced Fe_3_Al. Branagan *et al.* [28] observed similar phenomenon in cases of very hard materials in contact. Little material transfer is observed in Figure 15a at the lowest sliding speed; however, as the speed increases to 0.8 m·s^−1^ (Figure 15c), a dark layer (possibly partly oxidized) covers parts of the counterpart. It should be mentioned that almost no significant difference was found on the worn surface of the counterpart when sliding under different loads (*i.e.*, 3, 5 and 7 N). The wear mechanisms do not appear to be so sensitive to the applied load in the investigated range.

## 4. Conclusions

Unreinforced Fe_3_Al coatings and Fe_3_Al-TiB_2_ composite coatings with two different boride quantities (*i.e.*, 30 and 50 vol.%) were produced by the high-velocity oxy-fuel (HVOF) spray deposition on a steel substrate. The composite coatings consisted of a TiB_2_ phase, uniformly dispersed within a Fe_3_Al matrix. It was revealed that, by increasing the volume fraction of TiB_2_, both the Vickers hardness and sliding wear resistance of the coatings against an alumina counterpart (6.33 mm in diameter) were increased. The increase of wear resistance is believed to be related to the H/E value increase, which in turn is due to the presence of TiB_2_ particles within the Fe_3_Al matrix.

As the sliding speed increases, the dry sliding wear rate of the coatings increases to a maximum, after which the rate decreases as the sliding speed further increases. Adhesive and fatigue wear play important roles in the case of the unreinforced coating, while abrasive wear is most likely the predominant wear mechanism in the case of the reinforced coatings. Dry sliding at very high speeds resulted in partial oxidation of the surface due to high interfacial temperature. This leads to a reduction in the friction coefficient and wear rate.

## Figures and Tables

**Table 1:** HVOF spraying parameters.

Oxygen Flow Rate (SCFH)	Kerosene Flow Rate (GPH)	Carrying Gas	Spraying Distance (in)	Number of Deposition Passes
1500	6.2	Argon	15	10

**Table 2:** Microindentation hardness, reduced elastic modulus and *H*/*E* ratio of the coatings.

Sample	Vickers Hardness (H_v_)	*E*_r_ (GPa)	*H*/*E*
unreinforced Fe_3_Al	390.68	121.15	3.22
Fe_3_Al-30 vol.% TiB_2_	996.42	198.37	5.02
Fe_3_Al-50 vol.% TiB_2_	1252.71	201.15	6.22
